# Novel Implant Coating Agent Promotes Gene Expression of Osteogenic Markers in Rats during Early Osseointegration

**DOI:** 10.1155/2012/579274

**Published:** 2012-10-30

**Authors:** Kostas Bougas, Ryo Jimbo, Ying Xue, Kamal Mustafa, Ann Wennerberg

**Affiliations:** ^1^Department of Prosthodontics, Faculty of Odontology, Malmö University, 205 06 Malmö, Sweden; ^2^Department of Clinical Dentistry, Center for Clinical Research, Faculty of Medicine and Dentistry, University of Bergen, Årstadveien 17, 5009 Bergen, Norway

## Abstract

The aim of this study was to evaluate the early bone response around laminin-1-coated titanium implants. Forty-five rats distributed in three equally sized groups were provided with one control (turned) and one test (laminin-1-coated) implant and were sacrificed after 3, 7, and 21 days. Real-time reverse-transcriptase polymerase chain reaction was performed for osteoblast markers (alkaline phosphatase, runt-related transcription factor 2, osteocalcin, type I collagen, and bone morphogenic protein 2), osteoclast markers (cathepsin K and tartrate-resistant acid phosphatase), inflammation markers (tumor necrosis factor **α**, interleukin 1**β** and interleukin 10), and integrin **β**1. Bone implant contact (BIC) and bone area (BA) were assessed and compared to the gene expression. After 3 days, the expression of bone markers was higher for the control group. After 7 days, the expression of integrin **β**1 and osteogenic markers was enhanced for the test group, while cathepsin K and inflammation markers were down-regulated. No significant differences in BIC or BA were detected between test and control at any time point. As a conclusion, implant coating with laminin-1 altered gene expression in the bone-implant interface. However, traditional evaluation methods, as histomorphometry, were not adequately sensitive to detect such changes due to the short follow-up time.

## 1. Introduction

Dental implants have been proven to be a reliable long-term therapy against edentulism [1–3]. However, the reported high success figures of implant therapy have been based on implants inserted using two-stage surgical protocol and conventional loading. The increased demand on implant performance and the broadened treatment indications have led to the development of new moderately rough surfaces. Alterations in both the surface chemistry and topography may contribute to chemical influence on bone tissue, a phenomenon defined as bioactivity [4]. Furthermore, other factors such as surface energy, surface wettability, cellular maturation state, nutrition status, and microstresses alter the degree of bioactivity too. Compared to the previously used turned implants, the bioactively modified implants have demonstrated higher success rate in demanding cases, for example, early functional loading [5], one-stage surgery [6], and reconstructive jaw surgery [7]. 

When moderately rough surfaces remain within bone tissue no differences on microbial colonization are observed as compared to minimally rough surfaces [8]. However, there has been increasing evidence pointing out that as soon moderately rough implants are exposed to the oral milieu the case changes. A series of studies examining clinical, histological, and radiological aspects of experimental peri-implantitis in a dog model has reported that exposure of the implant surfaces to the oral environment leads to spontaneous progression of experimental peri-implantis [9–11]. The same research group has reported that implant surface characteristics affect the possibility to treat experimental peri-implantitis without antimicrobial therapy, thereby influencing the treatment outcome [12]. Additionally, a recent *in vitro* study has proposed that increased implant roughness promotes bacterial colonization most likely depending on protection of bacteria from shear forces [13]. Even if the referred studies are experimental in nature, the idea of developing an implant that combines the osseoconductive properties of a moderately rough surface with the accessibility for debridement of turned surfaces is intriguing.

In order to enhance bone formation, implants have been coated with bone specific biomolecules [14–17]. Interestingly, even non-bone-specific molecules have reported to induce osteogenicity [18]. One potential non-bone-specific osteogenic molecule is laminin-1. Laminins are heterotrimeric glycoproteins that bind to integrins, especially *β*1 and *β*2 isomers [19]. The N-terminal of laminin-1 has been reported to selectively recruit osteoprogenitors through integrin *β*1-mediated cell attachment [20, 21] and to stimulate production of alkaline phosphatase by osteoblasts [22]. Additionally, recent *in vitro* studies [23, 24] have elucidated the role of laminin as nucleation center and its potential to enhance osteoid formation in a simulated body fluid. Nevertheless, since the *in vivo* environment is more complex in terms of protein interactions [25] and desorption of the coating agent [26], *in vivo* validation has been imperative. In theory, any effects of a protein coating are more pronounced during the early stages of osseointegration. 

The purpose of this *in vivo* study is to investigate the detailed molecular mechanisms underlying the possible effects of the coating agent laminin-1 on osseointegration and to compare them to histological evaluation methods. 

## 2. Materials and Methods

### 2.1. Implants and Laminin-1 Coating

In total, 90 threaded titanium (grade 4) implants with turned surface were used (diameter: 1.5 mm, length: 2.5 mm, internal hexagonal connection, batch 800101579, Neodent, Brazil). Half of the implants (*N* = 45) were coated with laminin-1 in accordance with previous *in vitro* study [24] and served as the test group. In brief, laminin-1 (L2020, Sigma-Aldrich, Stockholm, Sweden) was diluted to a concentration of 100 *μ*g/mL in Dulbecco's phosphate-buffered saline (DPBS) without CaCl_2_ or MgCl_2_ (14190-094; GIBCO, Invitrogen Corporation, Grand Island, NY, USA). The implants were subsequently incubated in 48-well plates (Nunclon Surface, Nunc, Roskilde, Denmark) containing 250 *μ*L of the laminin-1 solution per well, for 1 h at room temperature. The protein thickness after incubation was estimated by ellipsometry. Since the implant surface did not reflect the light beam in a measurable manner, the amount of adsorbed laminin was calculated on optically smooth titanium surfaces produced at the laboratory as described by Linderbäck et al. [27]. As previously described by Bougas et al. [24], the optically smooth titanium surfaces were fixed in the ellipsometric quvette filled with PBS at room temperature. The ellipsometric angles Δ_0_ and Ψ_0_ were measured at three locations with a Rudolph Research AutoEL III ellipsometer operating in a wavelength of 632.8 nm at a 70° angle of incidence. Subsequently, the quvette was emptied and filled with laminin solution and new angles Δ and Ψ calculated. The protein layer thickness was calculated from the ellipsometric angle changes for a protein refractive index of *n* = 1.465. By using the McCrackin algorithm for the calculations [28], it was concluded that the incubation resulted in protein thickness corresponding to 2.6 nm. The remaining 45 uncoated implants served as controls.

### 2.2. Surface Characterization

The surface topography of the implants was characterized with an optical interferometer (MicroXam, ADE Phase Shift, Tucson, AZ, USA) operating in wavelength of *λ* = 550 nm. According to the proposed guidelines for implant surface characterization [29], three implants from each group were randomly selected and each measured in 9 regions (3 thread tops, 3 thread valleys, and 3 flank regions). A B-spline filter was applied to separate roughness from form and waviness. The following three topographical parameters were evaluated: an amplitude parameter, Sa (*μ*m) = the arithmetic average height deviation from the mean plane; a spatial parameter, Sds (*μ*m^−2^) = the density of summits; and a hybrid parameter, Sdr (%) = the developed surface ratio.

### 2.3. Animals and Surgical Procedure

The study was approved by the Malmö/Lund, Sweden, Regional Animal Ethical Committee (approval number: M253-10) and included 45 male Wistar Hannover Galas rats with an average weight of 350 g.

Prior to surgery, the animals were sedated by intraperitoneal administration of a mixture of Dormicum 5 mg/mL (Midazolam, Roche), Hypnorm (fentanylcitrate 0.315 mg/mL and fluanisone 10 mg/mL, Janssen Pharmaceutical) and sterile saline 0.9 mg/mL (Braun) in a dose of 1.5–2 mL/kg body weight. The hind legs were disinfected with 70% ethanol and 70% chlorhexidine, and Lidocaine hydrochloride (Xylocaine; AstraZeneca AB) was administrated as the local anaesthetic at each insertion site at a dose of 0.5 mL. One control implant was operated into the right tibia and one test implant into the left of each animal. One animal died after the sedation procedure. After the operation, buprenorphine hydrochloride (0.5 mL Temgesic; Reckitt Benckiser, Slough, UK) was administered as an analgesic for 3 days. 

### 2.4. RNA Extraction and Real-Time Reverse-Transcription Polymerase Chain Reaction

#### 2.4.1. Sample Retrieval

The animals were divided into three groups and were sacrificed after 3 days (*N* = 14), 1 week (*N* = 15) and 3 weeks (*N* = 15) with an overdose of carbon monoxide in a gas chamber. The skin above the implants was incised, and 20 implants (10 pairs control/test) for each of the two first groups (3 days and 1 week) were turned out manually. Since one implant in the 3-week group did not osseointegrate, 18 implants (9 pairs control/test) were turned out manually. The removed implants, along with the interface bone tissue, were placed in RNA*later *solution (QIAGEN GmbH, Hilden, Germany), and frozen at −80°C until analysis. 

#### 2.4.2. RNA Extraction from Implant Screws

The samples were processed in the TissueLyser instrument (Qiagen GmbH) together with *β*-mercaptoethanol RNeasy Lysis-buffer to remove and disrupt the cells attached to the surface of the implant screw. RNA was extracted from the sample mixtures with RNeasy Micro Kit number 74004 (Qiagen GmbH) according to manufacturer's instructions, including carrier to minimize decrease in yield due to small sample quantity. During extraction, all samples were DNase-treated according to manufacturer's instructions with RNase free DNase Set #1023460 (Qiagen GmbH) to reduce gDNA contamination.

#### 2.4.3. Reverse Transcription (RT)

All RNA samples were reverse transcribed in single 10 *μ*L reactions according to manufacturer's instructions using TATAA RT Kit number A103b (TATAA Biocenter AB) to generate cDNA. RT-controls were included to monitor the presence of gDNA. The controls were analyzed in pools of five, containing 1.5 *μ*L of each sample (total volume 7.5 *μ*L). Control of RNA concentration was not possible due to the presence of carrier in extraction procedure.

#### 2.4.4. Real Time Reverse-Transcription Polymerase Chain Reaction (Real Time RT-PCR)

For each Real Time RT-PCR, 10 *μ*L mixtures were prepared with 1 *μ*L cDNA, according to the manufacturer's recommendations (Applied Biosystem, CA, USA). Amplification was carried out in 96-well thermal cycle plates on a StepOne detection system (Applied Biosystems, USA) according to the manufacturer's recommendations with custom-designed real-time assays and SYBR green detection (PrimerDesign Ltd, Southampton, UK) (Table 1). Normalization and fold-changes were calculated with StepOneTM software with the ΔΔCt method [30].

### 2.5. Histomorphometry

The remaining implants from each group were retrieved *en bloc* and were immersed in 4% neutral buffered formaldehyde. Since one implant from the 3 week group did not osseointegrate possibly due to an incorrect insertion angle, the final numbers of implants processed for histology were; *n* = 8 for 3 days, *n* = 10 for 1 week, and *n* = 8 for 3 weeks. All the samples were processed for undecalcified ground sectioning [31]. Briefly, after a series of dehydrations and infiltrations in resin, the samples were embedded in light-curing resin (Technovit 7200 VLC; Heraeus Kulzer Wehrheim, Germany). One central ground section was prepared from each implant by using Exakt sawing and grinding equipment (Exakt Apparatebau, Hamburg, Germany). The sections were ground to a final thickness of approximately 10 *μ*m and histologically stained with Toluidine blue mixed with pyronin G.

Histological evaluations were performed using a light microscope (Eclipse ME600; Nikon, Japan), and the histomorphometrical data were analyzed by image analysis software (Image J v. 1.43u; National Institutes of Health, Bethesda, Maryland). The bone-implant contact (BIC) and the bone area (BA) percentage along the whole implant were calculated at ×10 objective magnification as described previously [32, 33].

## 3. Statistics

The statistical calculations were performed with SPSS (version 18 Chicago, Illinois, USA). The statistical comparison for the mean values of the topographic parameters Sa, Sds, and Sdr was assessed by Students *t*-test. For BIC and BA the nonparametric Wilcoxon signed ranks test was used while for relative gene expression, the Student's paired *t*-test was employed. The level of statistical significance was set at *P* ≤ 0.05.

## 4. Results

### 4.1. Surface Characterization

The laminin-1 coating increased the density of summits (Sds) significantly (*P* = 0.009). On the contrary, the protein coating did neither affect the average height deviation from the mean plane (Sa) (*P* = 0.261) nor the developed surface ratio (Sdr) (*P* = 0.446) of the implants significantly (Table 2). 

### 4.2. Real Time RT-PCR

**Figure 1:**
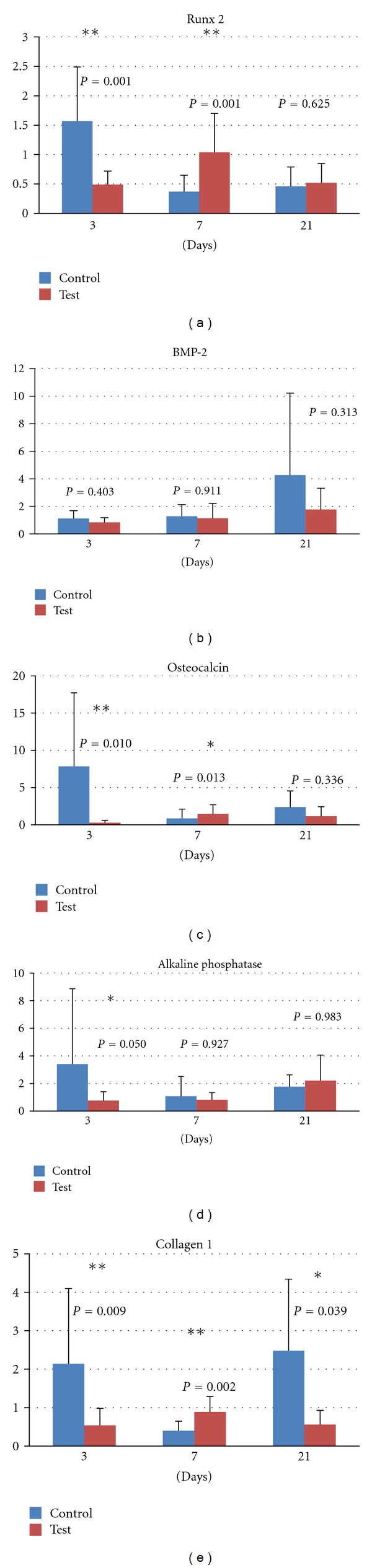
Relative gene expression for osteoblast markers at 3, 7, and 21 days: (a) runt-related transcription factor 2, (b) bone morphogenic protein 2, (c) osteocalcin, (d) alkaline phosphatase, and (e) type I collagen.

Although the gene expression for the osteoprogenitor marker runt-related transcription factor 2 (Runx2) was lower for the test than for the control after 3 days, it was doubled at 7 days resulting in statistically significantly higher levels as compared to the control. After 21 days, the difference in the expression of the gene for Runx2 between test and control was evened out (Figure 1(a)). The second osteoprogenitor differentiation, marker bone morphogenic protein-2 (BMP-2), did not differ between test and control at any time point (Figure 1(b)). 

The initial gene expression (3 days) of the osteblastic markers osteocalcin (Figure 1(c)), alkaline phosphatase (ALP) (Figure 1(d)) and type I collagen (Figure 1(e)) was higher for the control group. Nevertheless, after 7 days the expression of the osteoblastic markers increased for the test group and declined for the control. This alteration resulted in statistically significantly higher mRNA levels of osteocalcin and type I collagen in favour of the test group. After 21 days no statistically significant differences were detected between test and control in the expression of osteoclacin and ALP. On the contrary, the expression of type I collagen for the control group was enhanced to statistically significantly higher levels as compared to the test group. 

**Figure 2:**
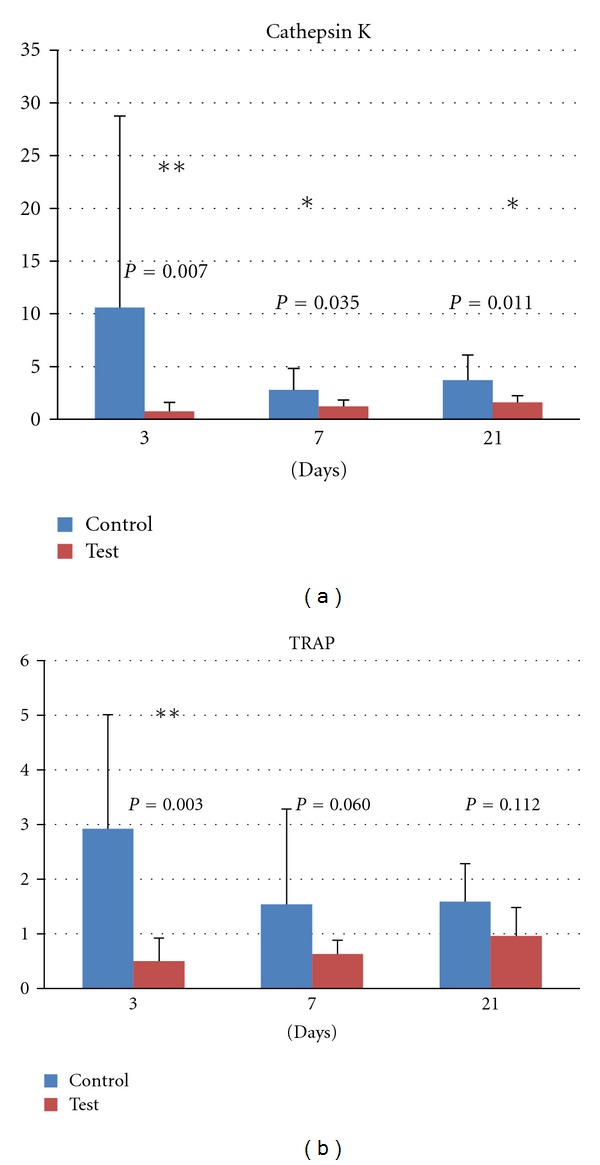
Relative gene expression for osteoclast markers at 3, 7, and 21 days: (a) cathepsin K and (b) tartrate-resistant acid phosphatase.

The expression of the osteoclastic marker Cathepsin K (Figure 2(a)) demonstrated a descending trend for the control group during the observation time. In contrast to the control group, the expression of Cathepsin K remained stable for the test group throughout the observation period. The expressed levels of Cathepsin K were statistically significantly lower for the test group at all times (3 days, 7 days, and 21 days). The mRNA levels of the osteoclastic marker tartrate-resistant acid phosphatase (TRAP) (Figure 2(b)) declined with time. After 3 days, the levels of TRAP were statistically significantly higher for the control group, whilst no statistically significant differences were detected between test and control at 7 or 21 days. 

**Figure 3:**
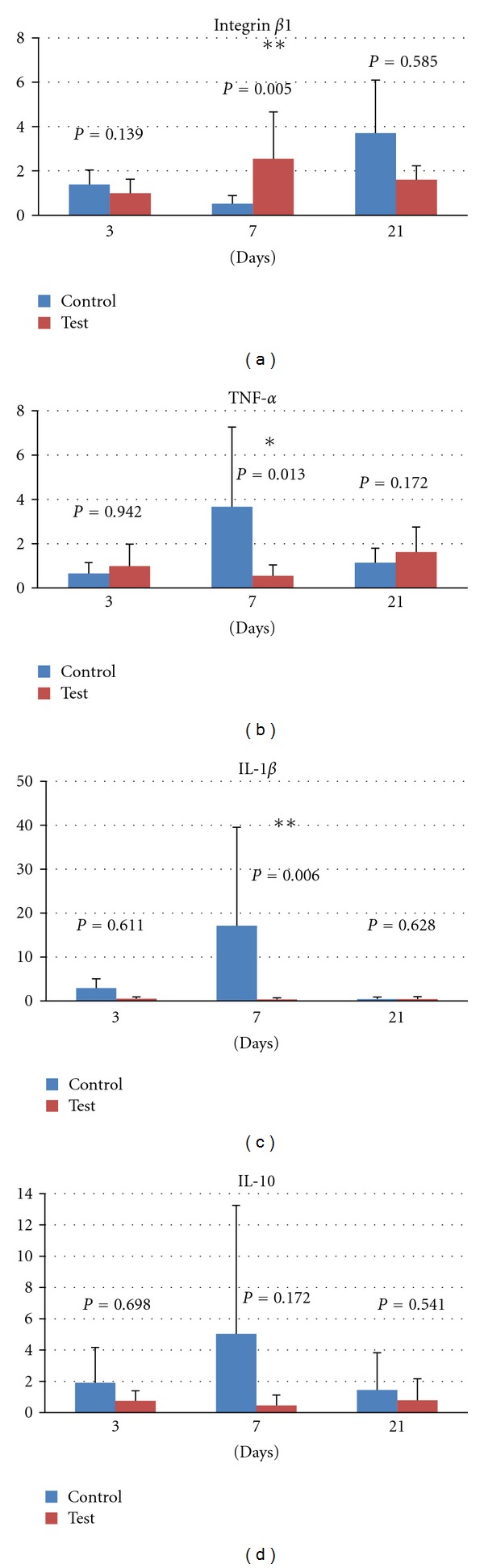
Relative gene expression at 3, 7, and 21 days: (a) integrin *β*1, (b) tumor necrosis factor *α*, (c) interleukin 1*β*, and (d) interleukin 10.

The gene expression of integrin *β*1 (Figure 3(a)) for the test group peaked at 7 days. At this time point, the mRNA levels for integrin *β*1 were statistically significantly higher for the test group (4.90-fold). Despite the enhanced levels of integrin *β*1 mRNA expression in the control group after 21 days, no statistically significant differences were detected. 

The proinflammatory cytokines tumor necrosis factor *α* (TNF-*α*) (Figure 3(b)) and interleukin 1*β* (IL-1*β*) (Figure 3(c)) peaked at 7 days. The fold of relative mRNA expression was 6.65 for TNF-*α* and 51.88 for IL-1*β* in favour of the control group. However, no statistically significant differences were detected at 3 and 21 days. The expression of TNF-*α* and IL-1*β* remained stable for the test group at 3, 7, and 21 days. No statistically significant differences were detected between test and control in the expression of the anti-inflammatory cytokine IL-10 (Figure 3(d)). 

### 4.3. Histomorphometry

**Figure 4:**
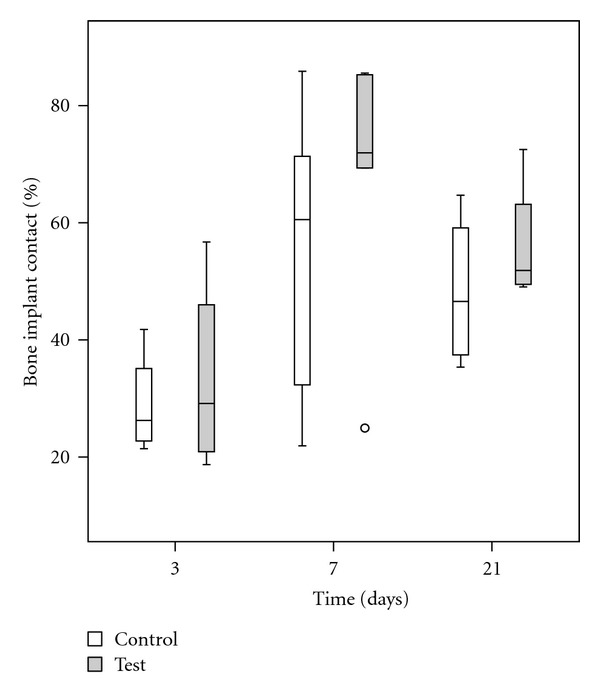
Box plot describing bone implant contact (%) at 3, 7, and 21 days.

**Figure 5:**
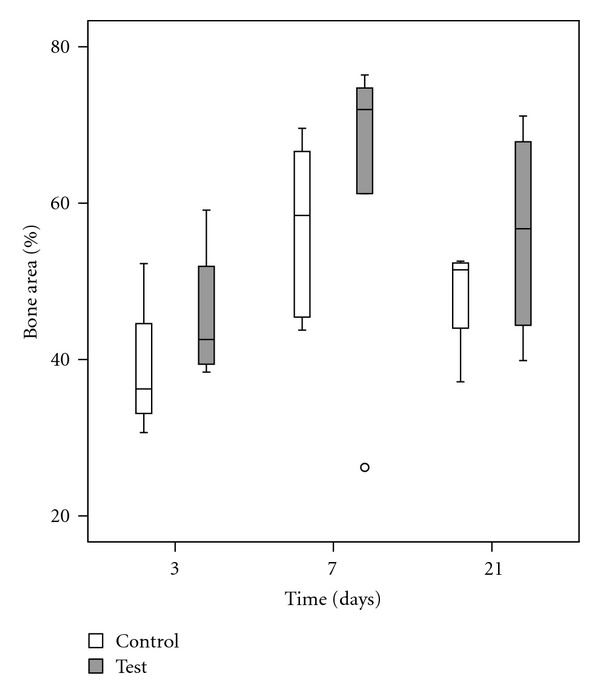
Box-plot describing bone area (%) at 3, 7 and 21 days.

The values of BIC and BA were enhanced after 7 and 21 days as compared to 3 days. The test group demonstrated higher median BIC and BA at all the evaluated time points (Figures 4 and 5). The differences between the test and control were though not statistically significant (*P* > 0.05) (Table 2). 

## 5. Discussion

In the current study, we have investigated the effects of laminin-1 coating on the early stages of osseointegration. The implants chosen for this purpose are turned with a smooth surface (Sa = 0.28 *μ*m). Implants with turned surface have a long history of clinical documentation [1–3] and hence may be regarded as the “gold standard.” The rational for rather using a smooth implant than a moderately rough, is based upon the theorem that moderately rough implants enhance osseointegration [34], thereby possibly concealing any effects of the biochemical coating. Additionally, a turned implant surface was chosen in order to investigate whether a biochemical coating can induce cell responses equivalent to the ones promoted by surface topography modifications [35, 36]. According to the surface analysis, the coating process has significantly increased the density of peaks (Sds). Since the implant surface has a minimally rough profile, the protein coating may be detected by the interferometer as prominences on the implant surface, hence resulting in elevated peak density of the test implant. 

Depending on protein-desorption kinetics [26], the biochemical coating is theoretically most active during the first days after the implant installation. For this reason, we have chosen to investigate the early effects of the laminin-1 coating after 3, 7, and 21 days. The early time-points investigated in this study have been previously used in the literature in order to screen the expression of bone-related genes and inflammatory markers in a rat model [35, 37]. Since the degree of diffusion of the coating into the surrounding bone is unknown, we have chosen to collect the interface bone tissue from the removed implant instead of retrieving the implant along with the surrounding bone *en bloc*. The analysis of the interface bone tissue is further justified by a histological study concluding that implant coating material released by the shear forces during implant insertion residues within the peri-implant space [38]. 

The gene analyses from the interface bone tissue reveal after 7 days significantly higher levels of the transcription factor Runx-2, which is the master gene for osteoblast differentiation and is expressed by the committed osteoprogenitors [39]. At the same time point, integrin *β*1 is significantly upregulated for the test implant. It has been suggested that laminin stimulates osteoprogenitors by attachment to integrin *β*1 *in vitro* [20, 21]. It has also been demonstrated that activation of Runx2 by MAPK is possible by binding of type I collagen to *α*2 *β*1 integrins [40]. Since collagen type I is also elevated after 7 days, a possible mechanism of action for laminin-1 could be indirect activation of Runx2 by elevating the expression of type I collagen which attaches to integrin *β*1. After 7 days, no significant difference in BMP-2 levels was detected between test and control. This may imply that laminin-1 has an indirect effect on the existing osteoprogenitors in the peri-implant space, without promoting commitment of the surrounding mesenchymal stem cells to osteoprogenitors via BMP-2 [41]. 

Apart from the molecular mechanisms involved in the effect of laminin-1 on osteoprogenitors, our results suggest an additional effect on differentiated osteoblasts. The gene expression of collagen type I and the marker for mature post-proliferative osteoblasts, osteocalcin [42], are up-regulated after 7 days. Hence, laminin-1 may contribute to the enrichment of the extracellular matrix in the direct proximity of the implant. If we additionally take into account the significantly decreased expression of the osteoclastic proteolytic enzyme cathepsin K [43], the bone remodeling [44] may be further displaced in favor of osteogenesis. As reported in the literature, low expression of cathepsin K can be correlated to low levels of the proinflammatory cytokines IL-1*β* and TNF-*α*, both considered to be expressed on bone sites with pronounced osteoclastic resorption [43]. As it concerns the 3-day time point, the expression of both osteogenic and osteoclastic markers is higher for the control group. Keeping into consideration that the outcome of the bone metabolism is decided by a coupled mechanism between bone deposition and bone resorption [44], it is uncertain whether this increased activity will result to more or less new bone. 

A comparison of the findings from the gene analyses to the findings from the histomorphometry reveals some discrepancies. Although there are important differences in gene expression, no differences are detected on BIC or BA after 3 and 7 days. This finding is considered reasonable keeping in mind that bone remodeling is a time-demanding process. Discrepancies between the results from traditional evaluation methods and genetic analysis have been reported previously [45]. The fact that no differences were detected on BIC or BA after 21 days is in agreement with the results from the gene expression. This result may be explained by the fact that the coating is expected to be more active during the early stages of osseointegration, since it may be gradually desorbed from the interface as demonstrated *in vitro* [26]. 

Conclusively, within the limitations of our study, we suggest that it is possible to alter the cell behavior on the implant-bone interface towards the osteogenic direction by coating the implant surface with laminin-1. However, the reported changes are not detected by histomorphometry, most likely depending on the fact that this method is not adequately sensitive at the short times of follow-up applied on the present study. 

## Figures and Tables

**Table 1:** Oligonucleotides used for real time RT-PCR.

Gene	Cat no.	AmpliconLength (bp)
Runx-2	Rn01512298_m1	86
ALP	Rn01516028_m1	68
Osteocalcin	Rn00566386_g1	104
BMP-2	Rn00567818_m1	126
Collagen 1	Rn01463848_m1	115
Integrin *β*1	Rn01753534_m1	82
IL-10	Rn00563409_m1	70
TNF-*α*	Rn99999017_m1	108
IL-1*β*	Rn00580432_m1	74
TRAP	Rn00569608_m1	95
CTSK	Rn00580723_m1	67
*β*-actin	4352931E	91

**Table 2:** Mean values (SD) for surface topography parameters for control and test implant, and *P*-values for pair-wise comparisons. Asterisk denotes statistically significant difference (*P* < 0.05).

Surface topography parameter	Control	Test	*P*-value
Sa (*μ*m)	0.284 (0.054)	0.280 (0.066)	0.261
Sds (*μ*m^−2^)	247493.49 (63993.65)	291112.17 (105683.8)	0.009*
Sdr (%)	14.38 (6.52)	20.16 (7.90)	0.446
